# Chloramine-induced anaphylaxis while showering: a case report

**DOI:** 10.1186/1752-1947-6-324

**Published:** 2012-09-25

**Authors:** Simona D’Alò, Tiziana De Pasquale, Cristoforo Incorvaia, Ilenia Illuminati, Gianni Mistrello, Daniela Roncarolo, Stefano Pucci

**Affiliations:** 1 Allergy Unit, General Hospital, Civitanova Marche, Italy; 2 Allergy/Pulmonary Rehabilitation Unit, ICP Hospital, Milan, Italy; 3 Research Department, Lofarma, Milan, Italy

## Abstract

**Introduction:**

Sodium-N-chlorine-p-toluene sulfonamide, commonly known as chloramine-T, is a derivative of chlorine which is widely used as a disinfectant. For many years, chloramine-T has been described as a cause of immediate-type hypersensitivity, especially with regard to asthma and rhinitis, and as a cause of occupational dermatoses in cleaning personnel in hospitals, although no anaphylactic reaction has yet been reported. Hence, to the best of our knowledge we present the first case of anaphylaxis to chloramine-T with evidence of specific immunoglobulin E antibodies.

**Case presentation:**

We describe the case of a 25-year-old Caucasian woman who was in good health and with a negative history for atopy, including no respiratory symptoms of rhinitis or asthma, and with no professional exposure to chloramine-T. She, while showering, applied a chloramine-T solution to a skin area with folliculitis on her leg, and within a few minutes developed generalized urticaria and angioedema, followed by vomiting and collapse with loss of consciousness. A skin prick test with a chloramine-T solution at 10mg/mL concentration was positive, and specific immunoglobulin E to chloramine-T was quantified at a value of 2.9 optical density as measured by the enzyme allergosorbent test technique.

**Conclusion:**

The strict cause-effect relationship and the results of the skin test and the *in vitro* test make certain the causative role of chloramine-T in this case of anaphylaxis. This suggests that chloramine-T, based on its wide use as a disinfectant, should be considered a possible cause in anaphylaxis of unknown origin.

## Introduction

Sodium-N-chlorine-p-toluene sulfonamide, commonly known as chloramine-T, is a derivative of chlorine with bactericidal and fungicide properties; it is widely used as a disinfectant. For many years, chloramine-T has been described as a cause of immediate-type hypersensitivity, especially asthma and rhinitis, with demonstration of specific immunoglobulin E (IgE) antibodies [1-3]. A recent survey on work-related asthma among health care professionals listed chloramine-T among the most frequent causative agents in the group of disinfectants [4]. Another clinical manifestation of allergy to chloramine-T is observed with respect to occupational dermatoses in cleaning personnel of hospitals [5], although thus far there is no report of anaphylactic reaction. To the best of our knowledge we present the first case of anaphylaxis to chloramine-T with evidence of specific IgE antibodies.

## Case presentation

The patient was a 25-year-old Caucasian woman in good health with a negative history for atopy, including respiratory symptoms of rhinitis or asthma, and with no professional exposure to chloramine-T. While taking a shower she applied a chloramine-T solution to a skin area with folliculitis on her leg and within a few minutes she had generalized urticaria and angioedema, followed by vomiting and loss of consciousness. On arrival at the Emergency Department her blood pressure was 80/50. The attending physician administered epinephrine 0.3mg intramuscularly, repeating the dose after a few minutes, methylprednisolone 1g intravenously and chlorphenamine 10mg intramuscularly, with a good clinical response. The patient was discharged from the hospital after 48 hours. We performed skin prick tests with a standard panel of inhalant and food allergens with negative results. However, a prick test with a chloramine-T solution at 10mg/mL concentration was positive, with a wheal diameter of 8mm. We chose this concentration because it was used in previous studies in patients with respiratory allergy to chloramine-T [1] and gave no positive result in control subjects. Blood was drawn by venipuncture for measuring specific IgE. For the *in vitro* test, polystyrene beads (6.4mm diameter, Precision Plastic Balls, Chicago, Illinois, USA) were coated with human serum albumin (HSA) according to the following procedure: Beads were pre-treated with 0.2M glutaraldehyde in phosphate buffered saline (PBS) for 5 hours at room temperature before being incubated overnight in end-over agitation with 150 mcL/bead of HSA (prepared at 6.6mcg/mL in PBS). After washings with PBS, beads were stabilized with 0.37% NaHBO_3_ in PBS for 1 hour before being saturated with 5% HSA in PBS for 24 hours; 50 HSA-activated beads were treated with chloramine-T according to Blomqvist *et al*. [3]. Briefly, beads were incubated with 20mL of 10mg/mL solution of chloramine-T in saline, after 2 hours of incubation the solution was replaced by 20mL of 10mg/mL of sodium pyrosulfite for 30 minutes at room temperature, and then beads were washed twice with saline and stored with 5% bovine serum albumin (BSA) in PBS until use.

Specific IgE were measured by the enzyme allergosorbent test technique as previously described [6]: briefly, 20mcL of the patient’s serum and 80mcL of PBS-BSA were added to HSA and to chloramine-HSA-coated beads and incubated overnight at room temperature. A negative control was performed using a pool of sera from non-allergic subjects. Bound-specific IgE were detected, after washing out the unbound fraction of serum, incubating for 2 hours with a peroxidase conjugated-goat anti-human IgE (BiosPacific, Inc., Emeryville, CA, USA) diluted 1:3000 in PBS-BSA. After further washings colorimetric reaction was developed (tetramethylbenzidine [TMB] conductivity 1, BioFX® Laboratories, Owings Mills, MD, USA) and stopped by the addition of 50mcL of 1M HCl. Finally 100mcL of each colored solution was transferred to empty wells and the absorbance read at 450nm using a Spectra ELISA (enzyme-linked immunosorbent assay) reader (SLT LabInstruments, Milan, Italy). The amount of specific IgE to chloramine-T was established on the basis of optical density (OD) values. The result was 2.9 OD for the patient's serum compared with 0.14 OD in the negative control serum.

## Discussion

Anaphylaxis is the most severe clinical expression of allergy and deserves continual investigation [7,8]. Most cases of anaphylaxis are caused by a few groups of agents, including drugs, foods, and insect venoms [8], but virtually any molecule can elicit this kind of allergic reaction. Discovering new agents is important because it reduces the need for a diagnosis of ‘idiopathic anaphylaxis’. Actually, such diagnosis weakens the management of patients by making an efficient prevention unfeasible. Sometimes, the newly discovered agent is never reported as a cause of allergy in general; sometimes the agent is known instead to be able to provoke other manifestations of hypersensitivity but not anaphylaxis. This is true for the case we describe. Chloramine-T is known as a cause of asthma and rhinitis, with demonstration of the IgE-mediated mechanism [1-3], and occupational dermatoses [5], but has not been described as a causal agent of anaphylaxis. The lack of previous reports prevented an immediate diagnosis based on a known cause and effect relationship, but the laboratory investigation demonstrated the responsibility of chloramine-T by detecting specific IgE antibodies. Interestingly, the patient had never had nasal or bronchial symptoms while previously using this disinfectant. It must be considered that the skin inflammation caused by folliculitis may have contributed to the reaction by favoring the absorption of chloramine-T.

## Conclusion

The strict cause-effect relationship and the results of the skin test and *in vitro* test make certain the causative role of chloramine-T in this case of anaphylaxis. This suggests that chloramine-T, based on its wide use as a disinfectant, should be considered a possible cause in anaphylaxis of unknown origin.

## Consent

Written informed consent was obtained from the patient for publication of this manuscript. A copy of the written consent is available by the Editor-in-Chief of this journal.

## Competing interest

The authors declare they have no competing interests.

## Authors’ contribution

SD and II collected the patient’s details and performed allergy tests. TD was a major contributor in writing the paper. GM and DR performed the laboratory investigation. SP was a major contributor in writing the paper. CI reviewed the paper and suggested changes. All authors read and approved the final manuscript.
